# Sugar sensing in C_4_ source leaves: a gap that needs to be filled

**DOI:** 10.1093/jxb/erae166

**Published:** 2024-04-20

**Authors:** Lily Chen, Oula Ghannoum, Robert T Furbank

**Affiliations:** ARC Centre of Excellence for Translational Photosynthesis, Hawkesbury Institute for the Environment, Western Sydney University, Hawkesbury Campus, NSW, 2753, Australia; ARC Centre of Excellence for Translational Photosynthesis, Hawkesbury Institute for the Environment, Western Sydney University, Hawkesbury Campus, NSW, 2753, Australia; ARC Centre of Excellence for Translational Photosynthesis, Research School of Biology, The Australian National University, Canberra, ACT, 2601, Australia; INRAE-Bordeaux, France

**Keywords:** C_4_ photosynthesis, hexokinase (HXK), Snf1-related kinase 1 (SnRK1), source and sink tissues, sugar sensing, target of rapamycin (TOR), trehalose 6-phosphate (Tre6P)

## Abstract

Plant growth depends on sugar production and export by photosynthesizing source leaves and sugar allocation and import by sink tissues (grains, roots, stems, and young leaves). Photosynthesis and sink demand are tightly coordinated through metabolic (substrate, allosteric) feedback and signalling (sugar, hormones) mechanisms. Sugar signalling integrates sugar production with plant development and environmental cues. In C_3_ plants (e.g. wheat and rice), it is well documented that sugar accumulation in source leaves, due to source–sink imbalance, negatively feeds back on photosynthesis and plant productivity. However, we have a limited understanding about the molecular mechanisms underlying those feedback regulations, especially in C_4_ plants (e.g. maize, sorghum, and sugarcane). Recent work with the C_4_ model plant *Setaria viridis* suggested that C_4_ leaves have different sugar sensing thresholds and behaviours relative to C_3_ counterparts. Addressing this research priority is critical because improving crop yield requires a better understanding of how plants coordinate source activity with sink demand. Here we review the literature, present a model of action for sugar sensing in C_4_ source leaves, and suggest ways forward.

## Teaser

C_4_ source leaf sugar sensing is underexplored compared with C_3_ plants. This review highlights advances in this area, focusing on C_4_ grasses and how this can be expanded upon.

## Introduction

Sugar sensing has emerged as a research topic of interest within the plant science community where mechanisms of action have predominantly been uncovered in the C_3_ model dicot *Arabidopsis thaliana*. Sugar sensing in plants was first proposed more than three decades ago when examining the metabolic regulation of gene expression in mesophyll protoplasts (Sheen, 1990). Despite intensive research on Arabidopsis, there has not been a transition into studying these mechanisms within cereals/monocots. Even though cereals such as wheat (*Triticum aestivum*), barley (*Hordeum vulgare*), maize (*Zea mays*), rice (*Oryza sativa*), and millets (e.g. Setaria spp.) make up a large proportion of the world’s food production, a huge knowledge gap exists on understanding how sugar sensing and signalling occur within these agronomically important crops (Godfray *et al.*, 2010; Lal, 2016; Springmann *et al.*, 2016; Simkin *et al.*, 2019). Some of the most productive plants on the planet, including many grasses and important crop species, utilize the C_4_ pathway of photosynthesis (Byrt *et al.*, 2011). Unlike C_3_ plants that fix CO_2_ directly from the atmosphere in the mesophyll cells using Rubisco, C_4_ plants have evolved a biochemical CO_2_-concentrating mechanism in which the enzyme phospho*enol*pyruvate carboxylase (PEPC) fixes atmospheric CO_2_ in the mesophyll cells to form a 4-carbon acid (C_4_ dicarboxylic acid). This C_4_ acid or its derivatives diffuses to the bundle sheath cells surrounding the vasculature and is decarboxylated to release CO_2_ where Rubisco is localized (Box 1A) (Hatch and Slack, 1966; Furbank, 2016). This two-cell system concentrates CO_2_ around Rubisco, reducing the wasteful process of photorespiration and ensuring Rubisco operates at its maximum catalytic capacity.

**Fig. 1.**
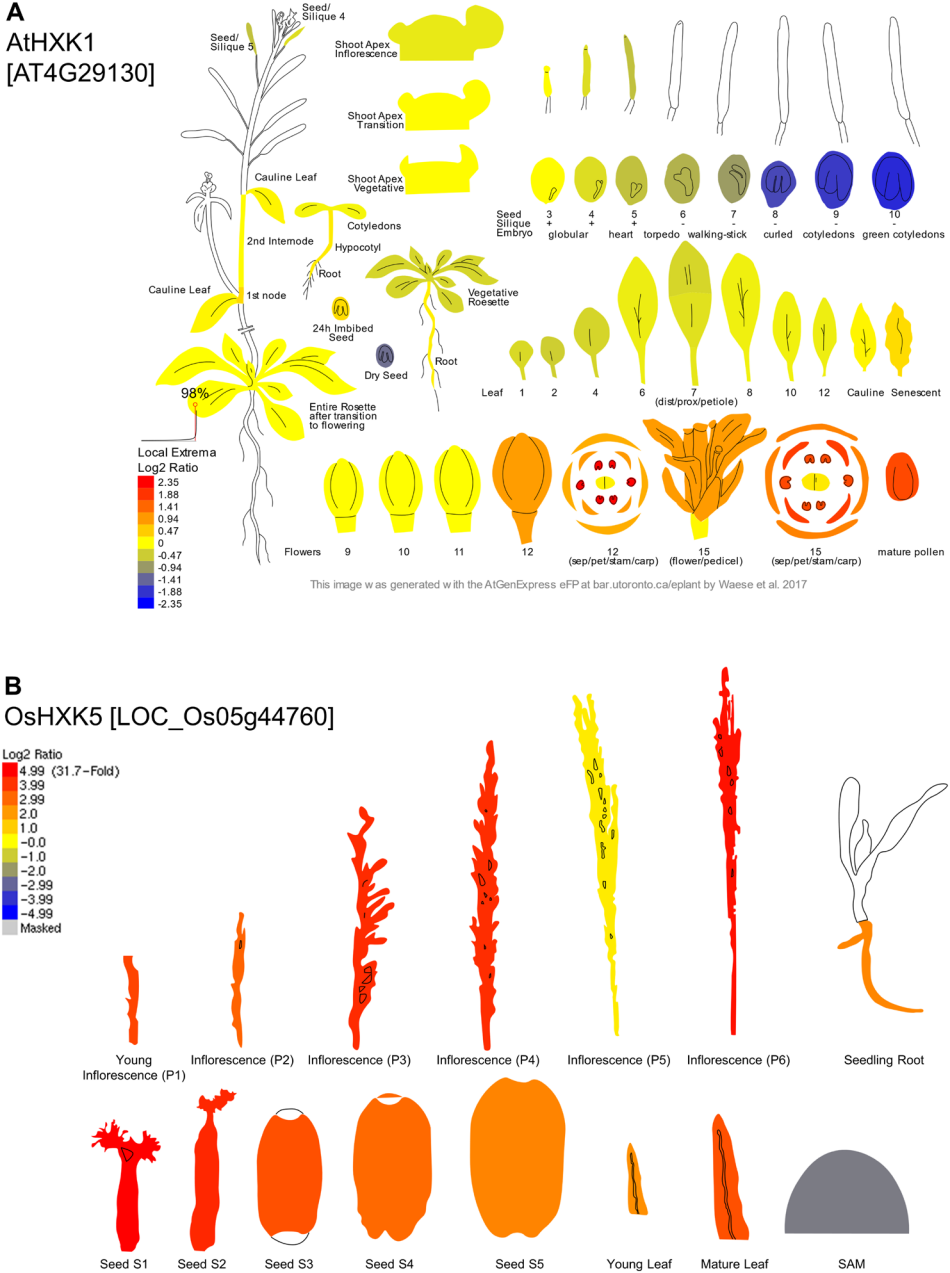

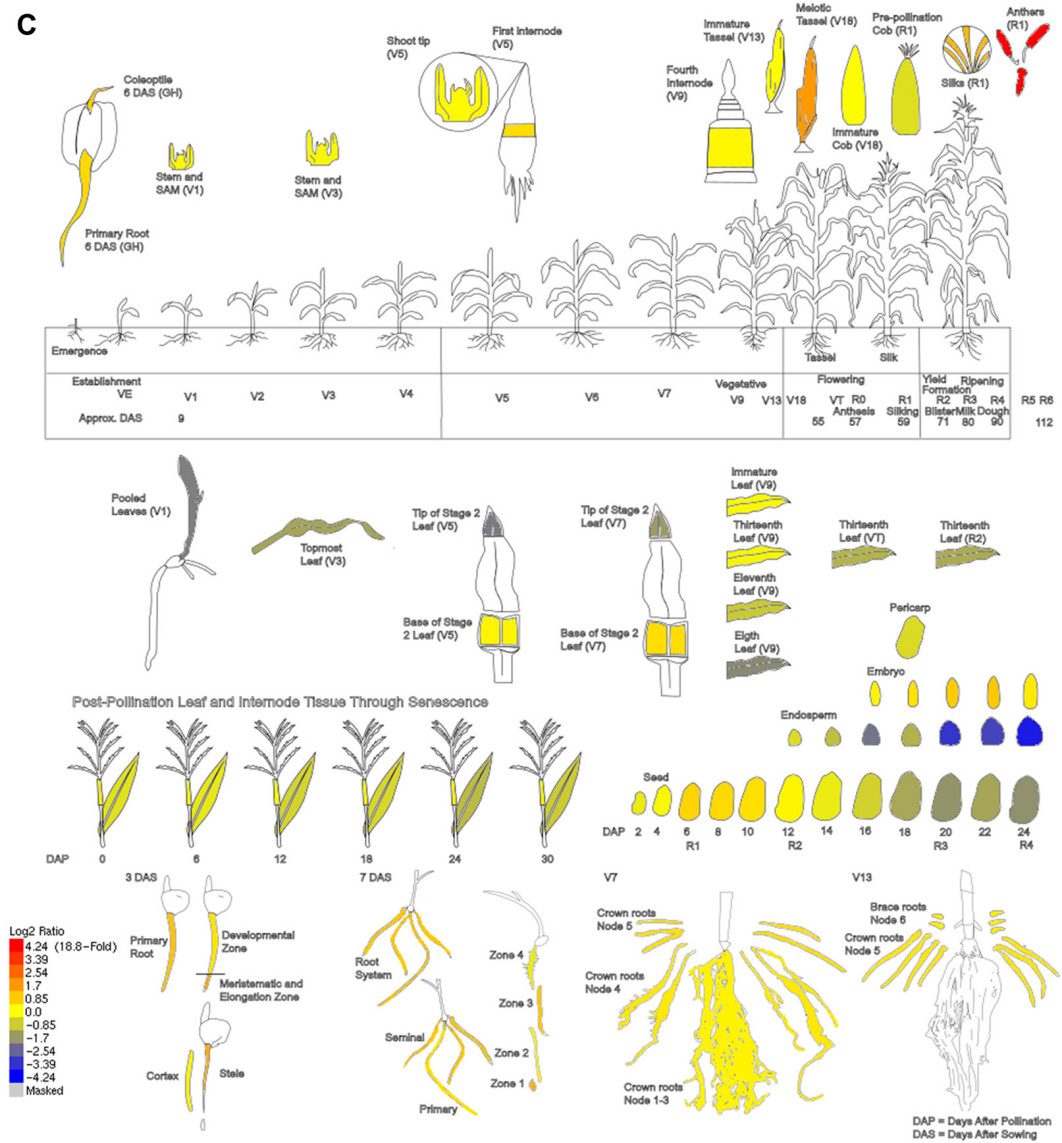
Electronic fluorescent pictograph (eFP) of sugar sensor hexokinase gene expression. The eFP depicts expression of the gene encoding the sugar sensor hexokinase in *Arabidopsis thaliana* (At) *AtHXK1* (A) (Nakabayashi *et al.*, 2005; Schmid *et al.*, 2005; Waese *et al.*, 2017), *Oryza sativa* (Os) *OsHXK5* (B) (Jain *et al.*, 2007), and *Zea mays* (Zm) *ZmHXK5* (C) (Hoopes *et al.*, 2019) across source and sink tissues. Expression is depicted as the log_2_ ratio where red, yellow, and blue represent high, moderate, and low expression, respectively. The gene name and ID are denoted in the top left corner. eFP figures were obtained from http://bar.utoronto.ca/ (Winter *et al.*, 2007).

The cellular specialization necessary for C_4_ photosynthesis also has implications for the regulation of carbohydrate synthesis and partitioning, and potentially for sugar signalling. In C_4_ plants utilizing the NADP-malic enzyme (NADP-ME) pathway of C_4_ photosynthesis (and possibly in all C_4_ plants), reduction of 3-phosphoglycerate (3PGA), required to regenerate ribulose 1,5 bisphosphate (RuBP) and also to generate 6-carbon sugar phosphates for starch and sucrose synthesis (Fig. 1), is spatially separated between the mesophyll and bundle sheath chloroplasts (von Caemmerer and Furbank, 2016). Up to two-thirds of the 3PGA diffuses from the Calvin cycle in the bundle sheath to the mesophyll chloroplasts and returns as glyceraldehyde 3-phosphate [G3P; also known as triose phosphates (TP)] (von Caemmerer and Furbank 2016; Furbank and Kelly, 2021). This diffusion requires a large concentration gradient with high levels of 3PGA present in the bundle sheath compartment and high G3P levels present in the mesophyll (Stitt and Heldt, 1985; Arrivault *et al.*, 2017). Since the transport of G3P out of the chloroplast and 3PGA and Pi levels inside the chloroplast are primary regulators of partitioning between sucrose and starch in C_3_ plants (McClain and Sharkey, 2019), this specialization has considerable ramifications for regulation of carbon partitioning in C_4_ leaves (Furbank and Kelly, 2021). In addition, in many C_4_ grasses, sucrose and starch synthesis are spatially separated, whereby sucrose is preferentially synthesized in the mesophyll compartment and starch in the bundle sheath chloroplast (see Box 1) (Lunn and Furbank, 1997, 1999; Furbank and Kelly, 2021). In some cases, this spatial specialization is achieved by transcriptional regulation of expression of key genes in sucrose biosynthesis (Furbank and Kelly, 2021). Global climate warming has drawn attention to C_4_ crops as they tend to be more climate resilient compared with their C_3_ counterparts. Since little attention has been paid to carbon partitioning and sugar signalling in leaves of C_4_ species, this review explores the impact of metabolic and cellular specialization in C_4_ leaves on these processes in this important group of plants.

Box 1. **Compartmentalization of photosynthesis and carbohydrate synthesis in C_4_ grass source leaves**

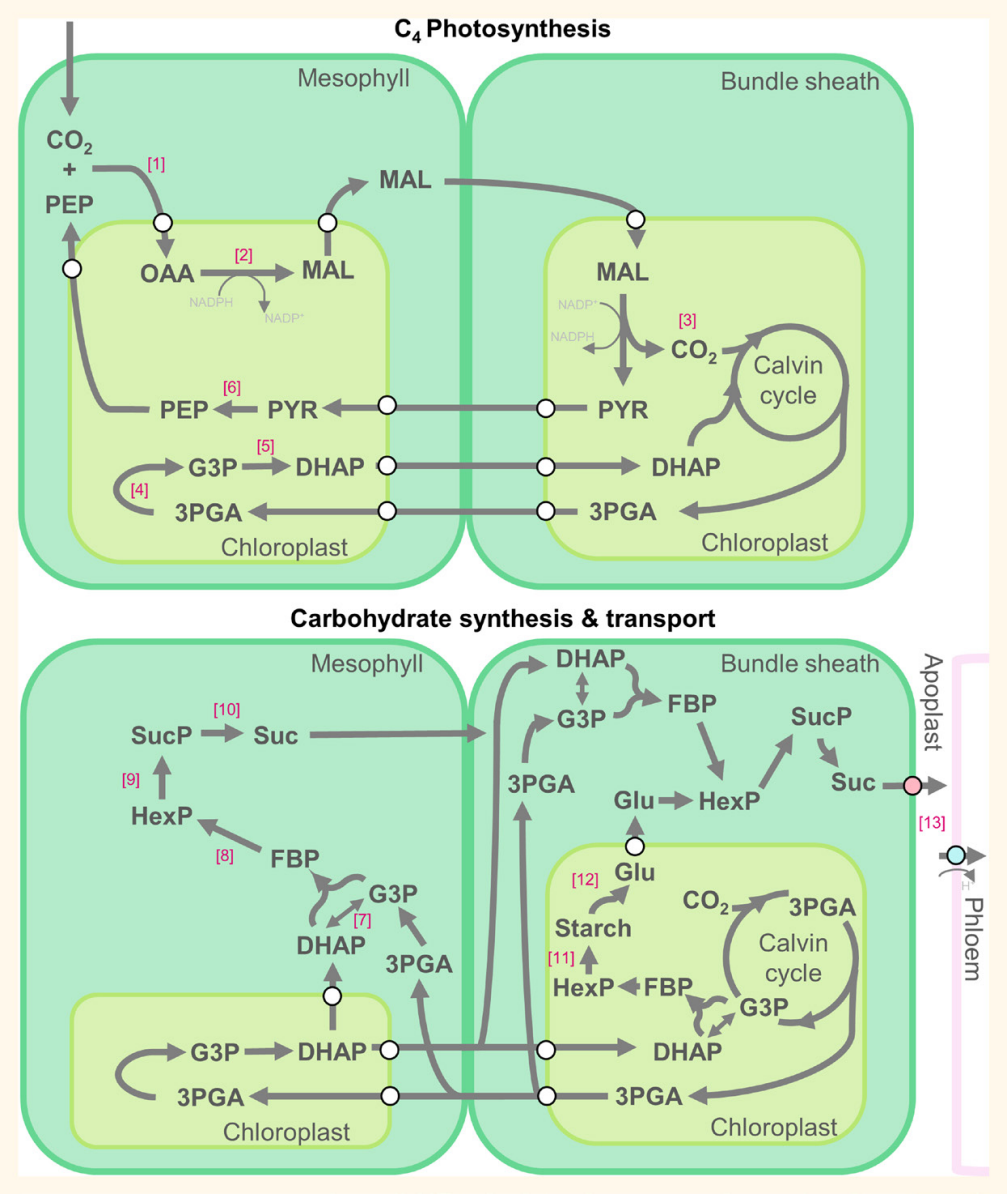

In the more common NADP-malic enzyme (NADP-ME) subtype of C_4_ plants, phospho*enol*pyruvate carboxylase (PEPC) catalyses the first carbon fixation step with phospho*enol*pyruvate (PEP) to form oxaloacetate (OAA) [1]. OAA enters the chloroplast via a plastidic transporter (white circle) where malate dehydrogenase (MDH) forms the C_4_ acid malate (MAL) [2]. MAL moves out of the chloroplast and diffuses across to the bundle sheath via the abundant plasmodesmata connections between the two cell types. MAL moves into the bundle sheath chloroplast where NADP-ME decarboxylates to release CO_2_ which enters the Calvin cycle to be refixed by Rubisco while also forming pyruvate (PYR) [3]. 3-Phosphoglycerate (3PGA) is formed in the Calvin cycle and can move back into the mesophyll chloroplast to form glyceraldehyde 3-phosphate (G3P) through reactions first catalysed by phosphoglycerate kinase (PGK) and then glyceraldehyde 3-phosphate dehydrogenase (GADPH) [4]. Triose phosphate isomerase (TPI) catalyses the reaction with G3P to form dihydroxyacetone phosphate (DHAP) [5] which can re-enter the Calvin cycle. Pyruvate phosphate dikinase (PPDK) catalyses the reaction with PYR shuttled from the bundle sheath to form PEP [6]. Sucrose and starch synthesis pathways of C_4_ grasses are unique compared with C_3_ dicots. 3PGA from the bundle sheath can be used in the mesophyll cytosol to form G3P from DHAP catalysed by TPI [7]. These substrates form fructose-1,6-bisphosphate (FBP) via fructose-1,6-bisphosphate aldolase (FBA). Hexose-phosphates (Hex-P) are formed from FBP through a series of reactions [8] to eventually result in sucrose-phosphate (Suc-P) via sucrose phosphate synthase (SPS) [9]. Sucrose phosphate phosphatase (SPP) cleaves Suc-P to form sucrose that can diffuse via plasmodesmata into the bundle sheath [10]. FBP in the bundle sheath chloroplast can be used to form Hex-P for starch synthesis via starch synthase (SS) [11] which is temporarily stored in the leaf and depleted through the night. When stored starch is needed, glucosidases break it down to glucose to be exported to the cytosol [12] and used for sucrose synthesis. Once sucrose reaches the bundle sheath it is exported by SUGARS WILL EVENTUALLY BE EXPORTED TRANSPORTERS (SWEET; pink circle), probably by SWEET13, into the apoplast for active uptake into the phloem tissues via sucrose transporter1 (SUT1; blue circle) [13]. Not all reactions are represented in this schematic for simplicity. The schematic is adapted from Furbank and Kelly (2021), where full pathways can also be found.

## Sugar transport and sensing in C_4_ photosynthesis

The implication of sucrose synthesis occurring preferentially in the mesophyll tissues of many of the important C_4_ crops is that sugars may move to the phloem for export via various routes. Sucrose could move from the mesophyll cells to the apoplast tissues and be reimported to the phloem sieve element–companion cell (SE–CC) complex (as is common in C_3_ plants) or sucrose could diffuse passively through the abundant plasmodesmatal connections between mesophyll and bundle sheath cells of the leaf (Danila *et al.*, 2016, 2018) and be exported to the phloem either symplastically or apoplastically in the bundle sheath cells. For the latter options, sucrose will need to move into the bundle sheath cells of C_4_ leaves down a concentration gradient which must be maintained between the two photosynthetic cell types (Turgeon and Ayre, 2005). The consumption of soluble sugars to produce starch in the bundle sheath cell would probably aid in maintaining this sucrose gradient. However, maintenance of high sugar levels in the mesophyll cells could potentially have a major impact on sugar perception and subsequent signalling pathways. Sucrose export pathways in C_4_ leaves have been more extensively elucidated from recent work on the passive membrane-embedded proteins SUGARS WILL EVENTUALLY BE EXPORTED TRANSPORTERS (SWEETs) which export photoassimilates out of the cell (Bezrutczyk *et al.*, 2021; Chen *et al.*, 2022; Hua *et al.*, 2022) and sucrose transporters (SUTs) which actively import sucrose into the SE–CC complex against a gradient from the apoplast (Box 1) (Aoki *et al.*, 2004; Carpaneto *et al.*, 2005). Uniquely, C_4_ plants must be able to coordinate carbon fixation within the highly metabolically active bundle sheath cell with temporarily housing hexoses for starch synthesis and providing a conduit for sucrose to be loaded into the phloem.

When sugars accumulate, such as under high light or high CO_2_ in some C_3_ species, synthesis can exceed the export capacity of these cells, especially when sink demand is low. If the accumulation of sugars is sensed in the photosynthetic cells, it triggers a signalling cascade inducing a down-regulation of photosynthesis by suppressing the expression of key genes encoding proteins in the photosynthetic pathway (for a detailed review of recent literature, see Fichtner *et al*., 2021). Although there are few data on sugar concentrations of individual photosynthetic cells in C_3_ or C_4_ species, it is possible that C_4_ species accumulate more sugars within these cells due to their increased photosynthetic efficiency. A pertinent question then is whether the coordination of photosynthesis with sink demand in C_4_ leaves occurs via mesophyll, bundle sheath, or both cells and how might the sugar sensors involved in this process have been adapted through evolution to accommodate the cellular specialization of C_4_ photosynthesis.

C_4_ photosynthesis has independently evolved at least 62 times over 60 million years (Sage *et al.*, 2011). During C_4_ evolution, pre-existing genes from C_3_ species were co-opted for altered functions in C_4_ species (Ludwig *et al.*, 2021). For example, of the two paralogues of the phospho*enol*pyruvate transporter (PPT), only PPT1 was co-opted to function in the C_4_ pathway, but not PPT2 (Lyu *et al.*, 2020). Gene duplication events have also been common in C_4_ evolution, including a key sugar transporter SWEET13 (Emms *et al.*, 2016). This study highlighted that the phloem loading strategies between C_3_ and C_4_ species might differ given the duplication of this transporter. Subsequently, many studies have since focused on this transporter and its role in phloem loading in C_4_ grasses such as sugarcane, Setaria, and maize (Bezrutczyk *et al.*, 2018, 2021; Hu *et al.*, 2018; Chen *et al.*, 2022). A recent analysis of protein sequences of known sugar sensors from the genomes of major groups of C_4_ grasses saw no positive selection of codons or duplication events during the evolution of C_4_ photosynthesis (Benning *et al.*, 2023). However, further studies on the evolution of sugar sensors and other proteins related to carbohydrate metabolism in C_4_ plants need to be expanded on.

## Sugar sensing and signalling in C_3_ species

Sugar sensing and signalling are usually studied either by artificially increasing sugars within the source leaves of plants through elevated CO_2_ or by exogenous sugar feeding. The relevance to climate change of crop responses to elevated CO_2_ means that this process has been thoroughly documented in free-air CO_2_ enrichment (FACE) experiments designed to better understand how ecological and plant systems respond to increased CO_2_ in the atmosphere (Leakey *et al.*, 2009). These FACE experiments usually used elevated CO_2_ between 550 ppm and 600 ppm or +200 ppm above ambient levels. In these conditions, some plants increased non-structural carbohydrates such as sugars and starches by between 30% and 40%, resulting in increased biomass (Ainsworth and Long, 2005; de Graaff *et al.*, 2006; Ainsworth, 2008). In the C_3_ crops soybean, wheat, and rice, the harvestable yield increased by 12–14% when grown in elevated CO_2_ (Long *et al.*, 2006; Ainsworth, 2008). Notably legumes and root crops have shown a greater increase in yield when compared with cereals (Ainsworth and Long, 2021). A comprehensive review of FACE experiments by Leakey *et al.* (2009) also observed that increased CO_2_ failed to meet predicted theoretical yield increases in many crop plants. This perhaps is due to not accounting for metabolic feedback of sugar accumulation within the leaves that causes suppression of photosynthetic activity through a range of transcriptional and post-translational processes (Sheen, 1990; Moore *et al.*, 2003; Oszvald *et al.*, 2018; Paul and Eastmond, 2020; Fichtner *et al.*, 2021). This has been demonstrated in a range of species under elevated CO_2_, with varying magnitudes of effects. In wheat, an increase in sink strength could not overcome photosynthetic acclimation and down-regulation under high CO_2_ when photoassimilates accumulated in the leaves (Aranjuelo *et al.*, 2011). Elevated CO_2_ over prolonged periods saw similar results in soybean and Arabidopsis, but the effect varied across ecotypes (J.-Y. Li *et al.*, 2008; Zheng *et al.*, 2019).

Many studies on high CO_2_ have also highlighted the importance of the nexus between carbon and nitrogen availability. During photosynthetic acclimation, insufficient nitrogen is being acquired and assimilated at high CO_2_, which in turn causes nitrogen limitation and a lower CO_2_ assimilation (P. Li *et al.*, 2008; Taub and Wang, 2008; Zheng *et al.*, 2019). The carbon to nitrogen sensing mechanism allows plants to efficiently modulate carbon and nitrogen transport and metabolism depending on the energy status of the plant. Therefore, when carbon is abundant and internal nitrogen is low, nitrogen assimilation genes can be activated, and conversely be halted when photoassimilate availability is low and internal nitrogen is high (Coruzzi and Zhou, 2001). For many C_3_ plants, yield responses can only be maintained if nitrogen is abundantly available (Ainsworth and Long, 2021).

Apart from imposing high CO_2_ conditions, sugar feeding on leaves excised from the main plant can also artificially increase sugar within source leaves. During glucose feeding of spinach (*Spinacia oleracea*) leaves, a marked decrease in key photosynthesis proteins such as Rubisco was observed, subsequently decreasing photosynthetic activity (Krapp *et al.*, 1991). Interestingly, a comparative study on the C_3_ dicot tobacco *(Nicotiana tabacum*) and C_4_ dicot *Flaveria bidentis* demonstrated that when exogenous sucrose was supplemented in the growth medium, it stimulated maximal photosynthetic rates comparable with when plants are grown in glasshouse conditions (Furbank *et al.*, 1997). It must be noted that in the latter study by Furbank *et al.* (1997) nitrogen was not limited, unlike in Krapp *et al.* (1991). These highly controlled experiments provided evidence for the link between photosynthesis and sugar sensing and signalling in C_3_ species, underlining the complexities of this pathway (Burnett, 2019).

A key sugar sensor that was first identified in Arabidopsis was HEXOKINASE1 (HXK1) (Moore *et al.*, 2003). AtHXK1 phosphorylates hexoses but also senses glucose within the leaves, where an increase in glucose caused a decrease in expression of photosynthesis-related genes such as *RbcS* which encodes the small subunit of Rubisco. Similar mechanisms have been documented in other C_3_ species such as tobacco (*N. tabacum*), rice, and potato (*Solanum tuberosum*) (Veramendi *et al.*, 2002; Cho *et al.*, 2009; Kim *et al.*, 2013), where homologues in monocots are usually HXK5 and HXK6. The relationship between sugar sensing and photosynthesis has been most well documented with hexokinase, but there has been evidence for other sugar sensors as well. For example, photosynthesis was altered when manipulating the trehalose synthesis pathway, more specifically by changing the levels of the sensing metabolite trehalose 6-phosphate (Tre6P) which acts as a proxy for sucrose levels (Pellny *et al.*, 2004;  Yadav *et al.*, 2014). Photosynthesis-derived glucose has been shown to modulate target of rapamycin (TOR) signalling, changing the transcriptome to regulate plant growth (Xiong *et al.*, 2013).  TOR forms part of a larger complex known as TOR complex 1 (TORC1) that also includes the regulatory proteins regulatory-associated protein of TOR (RAPTOR) and Lethal with Sec Thirteen 8 (LST8). Snf1-related protein kinase1 (SnRK1) has been shown to have a role in the starvation response in plants when photosynthesis is limited by up-regulating catabolic processes (Baena-González *et al.*, 2007). SnRK1 comprises catalytic kinase α subunits and regulatory β, γ, and βγ subunits, the latter of which are specific to plants.

Under conditions of sugar build up in source leaves, glucose can act as a potent inhibitor of photosynthetic gene expression in plants. However, sucrose, fructose, and more recently Tre6P have also been implicated as key signalling molecules, but sugar-specific signalling can be difficult to separate from a role in central metabolism (Cho and Yoo, 2011; Ponnu *et al.*, 2011; Li and Sheen, 2016; Baena-González and Lunn, 2020; Yoon *et al.*, 2021). Expression of invertase within different cell compartments such as the apoplast, cytosol, and vacuole of the source leaves has shown that sucrose is not responsible for regulating photosynthesis since it is down-regulated when hexoses accumulate in the photosynthetic cells after sucrose hydrolysis (Sonnewald *et al.*, 1991; Heineke *et al.*, 1992; Büssis *et al.*, 1997; Kingston-Smith *et al.*, 1999). Sugar sensors can form complexes or post-transcriptionally regulate proteins by modifying the overall transcription of genes. Under high glucose, HXK1 can interact with the vacuolar H^+^-ATPase B1 (VHA-B1) and the 26S proteasome AAA-ATPase subunit, specifically the Regulatory Particle 5b (RPT5B), to form a nuclear complex that regulates transcription (Cho *et al.*, 2006). TORC1 is known as a master regulator, where TOR phosphorylates ETHYLENE-INSENSITIVE 2 (EIN2) or PIN-FORMED 2 (PIN2), usually mediating processes associated with plant growth such as cytokinesis and cell elongation and expansion (Yuan *et al.*, 2020; Fu *et al.*, 2021). Conversely, SnRK1 usually suppresses energy-demanding processes during the starvation response. This complex phosphorylates C group basic leucine zipper 63 (bZIP63) affecting dimerization with S1 group bZIPs, which results in transcriptional changes (Mair *et al.*, 2015; Pedrotti *et al.*, 2018; Han *et al.*, 2020; Muralidhara *et al.*, 2021). Arabidopsis plant extracts from young seedlings showed that Tre6P suppresses SnRK1 activity, but not in extracts from mature leaves (Zhang *et al.*, 2009; Li *et al.*, 2021). The authors noted that the intermediary factor present in young seedlings was not present in mature tissues that would cause Tre6P inhibition of SnRK1. Tre6P and/or the sucrose:Tre6P ratio is critical for controlling carbohydrates during different developmental stages, as well as detecting nitrogen status through changes in sucrose levels (Schluepmann *et al.*, 2003;  Wahl *et al.*, 2013;  Yadav *et al.*, 2014). Similarly, it is possible that SnRK1 activity corresponds to tissue types and stage of growth in plants where in mature leaves anabolic pathways might be less active when compared with younger tissues. Although sugar sensing is already difficult to study in C_3_ species, this is even more complicated in C_4_ species (for more detail about the pathways, see Box 2 and references therein).

Box 2. **Schematic of sugar sensing/signalling pathways**

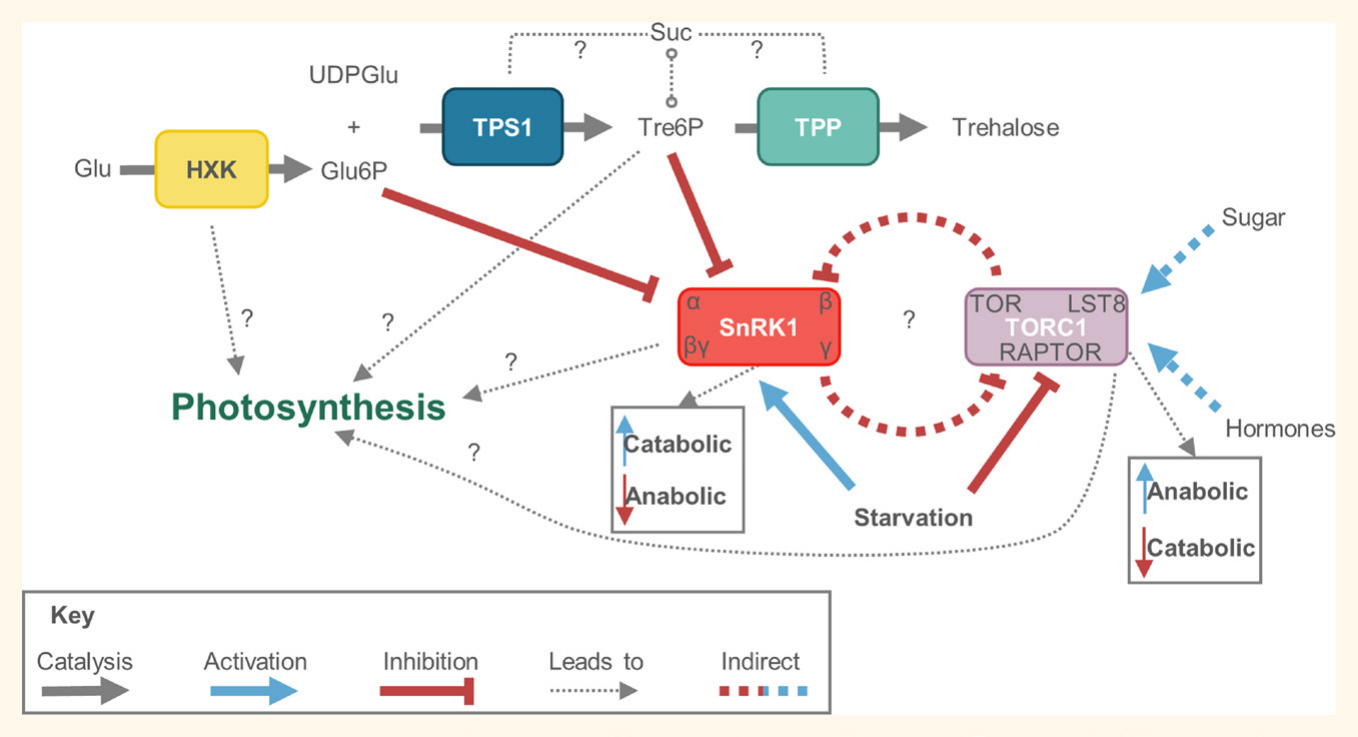

What researchers know about the sugar sensing/signalling pathway is derived from studies in Arabidopsis, the C_3_ dicot model species. Hexokinase (HXK), Snf1-related kinase 1 (SnRK1), and Target of Rapamycin complex 1 (TORC1) are known proteins/complexes involved in the sugar sensing/signalling pathways along with the sensing metabolite trehalose 6-phosphate (Tre6P) (Li *et al.*, 2021). HXK catalyses the phosphorylation of hexoses, mainly glucose (Glu), to form glucose 6-phosphate (Glu6P), and can also sense glucose independently from its catalytic function, usually HXK1 in dicots or HXK5 and HXK6 in monocots (Moore *et al.*, 2003; Cho *et al.*, 2009). Trehalose phosphate synthase 1 (TPS1) catalyses the reaction between UDP-glucose (UDPGlu) and Glu6P to form trehalose 6-phosphate (Tre6P), a sugar signalling metabolite which can indicate the sucrose (Suc) status (Paul *et al.*, 2020). Trehalose phosphate phosphatase (TPP) cleaves Tre6P to form trehalose (made up of two glucose molecules) which is only detected at very low amounts in plants. It has been established that a Suc–Tre6P nexus exists that can regulate metabolic pathways, but the specific mechanisms behind this remain ambiguous (Zhang *et al.*, 2009; Yadav *et al.*, 2014). It is hypothesized that there is a relationship between Suc and TPS1 activation, but it is unknown how this occurs and if there is also a direct link between Suc and TPP activity. Tre6P is a negative feedback regulator of Suc, modulating its levels in leaves to increase anabolic processes which can occur by suppressing SnRK1 activity (Baena-González and Lunn, 2020). Suppression of SnRK1 activity can also occur through Glu6P. During plant starvation, SnRK1 activity is increased, increasing catabolic (ATP-producing) processes but decreasing anabolic (ATP-consuming) processes. In contrast, TORC1 activity is decreased during starvation since its function usually increases anabolic and decreases catabolic processes (da Silva *et al.*, 2021). Although still unclear, sugar metabolism activates TORC1 alongside certain hormones such as auxin. While not discussed in detail here, SnRK1 comprises catalytic kinase α subunits and regulatory β, γ, and βγ subunits, the latter of which is specific to plants. TORC1 comprises the TOR kinase interacting with Regulatory-Associated Protein of TOR (RAPTOR) and the Lethal with Sec Thirteen 8 (LST8) proteins. The sugar metabolism pathway and these sensors have been implicated in regulating photosynthesis, but the mechanisms behind their role remain ambiguous. Furthermore, information on whether sugar sensing or signalling is ubiquitous or unique between the bundle sheath mesophyll cells of C_4_ species has been limited. Sugar sensing and signalling is difficult to study given the complexities of sensing and signalling pathways (see Fichtner *et al.*, 2021 and references therein for more detail), transduction cascades, and hormones involved in a C_3_ plant, but is further complicated in a C_4_ species where photosynthesis and sucrose and/or starch synthesis is compartmentalized, requiring orchestration between two cell types.

## Sugar sensing pathway in C_4_ species

Sugar sensing studies in C_4_ species have so far produced conflicting evidence about the role of sugar sensors and their relationship with photosynthesis. In contrast to C_3_ plants, many C_4_ species in FACE experiments did not experience an increase in net photosynthesis (Leakey *et al.*, 2009; Ainsworth and Long, 2021). For example, sorghum did not increase photosynthesis rates under high CO_2_ but did increase its carbon gain when subjected to drought by improving water relations due to enhanced photosynthetic performance at lower stomatal conductance under high CO_2_ compared with ambient levels (Wall *et al.*, 2001). FACE experiments with the C_4_ grass *Paspadalum dilatatum* or the major crop maize also did not show a net increase in photosynthesis (von Caemmerer *et al.*, 2001; Leakey *et al.*, 2004, 2006). The paradigm is that in elevated CO_2_, photosynthetic rates increase marginally or not at all in C_4_ species since the C_4_ photosynthetic mechanism has evolved to saturate Rubisco with CO_2_ within the bundle sheath cells at ambient mesophyll CO_2_ concentrations (Hatch and Osmond, 1976; von Caemmerer and Furbank, 2003). Many C_4_ species are only more productive under high CO_2_ when droughted and stomatal closure occurs to conserve water that evaporates during transpiration (Ainsworth and Long, 2021). As discussed earlier, C_3_ species increase photosynthetic rates due to the increased availability of CO_2_ within leaf mesophyll cells where Rubisco is located (Grodzinski *et al.*, 1998; Moore *et al.*, 1999; Ainsworth and Rogers, 2007). Since C_4_ species already operate at ‘CO_2_ saturation’ under ambient CO_2_ concentrations, it is possible that either they can bypass sugar feedback regulation of photosynthesis by not allowing photoassimilates to accumulate through cell type compartmentalization, by exporting it rapidly, or they are less sensitive to sugar increases within these cells. Interestingly, a 20 year FACE experiment has shown a reversal of the C_3_ and C_4_ trends in grasses under elevated CO_2_ in the latter 8 years where C_3_ grasses decreased and C_4_ grasses increased in total biomass (Reich *et al.*, 2018). This study highlighted that prediction of long-term results using currently known short-term drivers of plant responses could still be unreliable. Sugar sensing and signalling mechanisms may not hold true during prolonged exposure to elevated CO_2_ where long-term adaptation to increases in photoassimilates may occur.

Conflicting evidence in elevated CO_2_ conditions for C_3_ and C_4_ plants arises due to different species, CO_2_ levels, temperature, water/nutrient availability, light, and experimental conditions (e.g. pot size, duration of measurements), contributing to varying conclusions. A meta-analysis of biomass and photosynthetic rates found that it generally increased in C_3_ and C_4_ species but usually at a higher percentage for C_3_ plants. In *Amaranthus edulis*, a C_4_ dicot species, it was shown that elevated CO_2_ did not affect photosynthetic rates or carbohydrate accumulation (Blechschmidt-Schneider *et al.*, 1989). However, after cold treatment which inhibits sucrose translocation, rates of photosynthesis rapidly declined. This suggests that in this C_4_ species at least, feedback regulation of photosynthesis still occurs but the threshold at which this occurs might be different to C_3_ species.

In elevated CO_2_ conditions, sugarcane (*Saccharum* spp.), a major C_4_ grass crop, was found to accumulate 29% more sugars in the leaf than under ambient conditions, with an up-regulation of photosynthesis and its associated genes between the 13th and 22nd week of a 50 week experiment (De Souza *et al.*, 2008). Total biomass also increased by 40% compared with ambient conditions and, unlike observations in other C_4_ grasses, the sugarcane exhibited these trends under well-watered and fertilized conditions. In the C_4_ model grass species *Setaria viridis*, the down-regulation of photosynthesis was not observed under high light (1000 µmol m^−2^ s^−1^) even though there was high accumulation of sugars compared with medium light (500 µmol m^−2^ s^−1^). Transcriptional changes related to photosynthesis and sugar sensor genes were stronger under low light (50 µmol m^−2^ s^−1^) than high light (Henry *et al.*, 2020). These findings appear to contradict previous results established in C_3_ species where an increase in sugars down-regulates photosynthesis gene expression (Moore *et al.*, 2003; Cho *et al.*, 2009). This trend was also observed in isolated maize mesophyll cells when applying sucrose and glucose exogenously (Sheen, 1990). Sucrose concentrations >300 mM were shown to significantly decrease the promoter activity of key photosynthesis genes, pyruvate, phosphate dikinase (*PPDK*), *PEPC*, and *RbcS* in maize protoplasts when compared with normal levels of sucrose (30 mM) (Gerhardt *et al.*, 1987). The author postulated that photosynthetic regulation by sugars may only occur above certain physiological limits. Therefore, it is possible that although no down-regulation of photosynthesis was detected in some studies on C_4_ species despite the accumulation of sugars, the threshold to cause this repression of genes had not been reached.

Sugarcane is a major C_4_ grass crop and widely cultivated across the world due to its ability to store substantial amounts of sugars within its stems. During sugarcane phloem loading perturbation, it was found that photosynthesis rates and photosynthetic enzyme activity decreased across 5 d (McCormick *et al.*, 2008b, c). Similar findings have been uncovered when increasing sugars within the leaf by other methods such as exogenous application of sucrose and in elevated CO_2_, but with the latter decreasing assimilation only after a long period of exposure (De Souza *et al.*, 2008; Lobo *et al.*, 2015). Sugar accumulation within the leaf is an early symptom (prior to yellowing) of Yellow Canopy Syndrome (YCS) in sugarcane and can be used as a system to study effects of feedback regulation on photosynthesis (Marquardt *et al.*, 2019, 2021). Sugarcane suffering from YCS displays an accumulation of sugars in the leaf that precedes yellowing and a decrease in photosynthesis. RbcS and Rubisco activase (RCA) as well as PEPC transcripts and protein were not down-regulated in early-stage sugar accumulation. This was similarly observed with *RbcL* transcript except that the protein which it encodes was up-regulated early in sugar accumulation, perhaps suggesting some post-transcriptional modifications of *RbcL*. Early-stage accumulation of sugars could be attributed to the apoplast rather than the cytosol of photosynthetic cells as the infection of pathogens can often lead to an increase in apoplastic sugars. This response differs for different pathogens where for viruses they replicate within the cells unlike fungal and bacterial infections that usually access sugars via the apoplast (Liu *et al.*, 2022). The apoplastic and cytosolic sugars were not measured under YCS infection and therefore changes cannot be attributed to an increase of intracellular sugars specifically and perhaps this accounts for the absence of transcriptional and proteomic changes related to Rubisco and PEPC (Marquardt *et al.*, 2021). Many transcripts and proteins were only down-regulated during late-stage sugar accumulation, perhaps suggesting that a certain level of sugars might need to be reached within the cytosol before a down-regulation of photosynthetic genes occurs.

Perturbations between the source and sink by shading every leaf except one in sugarcane increased sink demand and suppressed the expression of genes encoding HXK sensor homologues, similar to results reported for *Setaria* (McCormick *et al.*, 2008a; Henry *et al.*, 2020). Disturbance of the source–sink balance between the only unshaded leaf in sugarcane and the subtending internode resulted in an increase in photosynthetic rate and its related genes. The authors suggested that this supported the notion that the sugar sensing HXK can regulate photosynthesis gene expression since decreased HXK sensing would result in less photosynthetic repression. The trehalose metabolism pathway has also been implicated in the sugar sensing mechanisms in sugarcane. Sugar accumulation in the leaves caused genes encoding trehalose 6-phosphate phosphatase (TPP) and trehalose 6-phosphate synthase (TPS) to be up- and down-regulated, respectively (McCormick *et al.*, 2008b). Previous evidence in the C_3_ dicot tobacco showed that, when *Escherichia coli* homologues of TPP and TPS were overexpressed, they increased and decreased photosynthesis, respectively (Pellny *et al.*, 2004). Overexpressing the same TPP and TPS homologues from *E. coli* in sugarcane increased and decreased sucrose levels, respectively (Gabriel *et al.*, 2021). Therefore, it is possible that similar mechanisms of sugar sensing via Tre6P signalling might be involved in regulating expression of photosynthesis genes in sugarcane and other C_4_ grasses. The importance of the Tre6P signalling pathway in C_4_ plants has recently been summarized by Rojas *et al.* (2023) and underlined the need to move knowledge beyond the model C_3_ dicots Arabidopsis and tobacco given their different physiology and anatomy as well as source–sink demands.

## A better understanding of C_4_ plant sugar sensing and signalling

In C_4_ plants, studying the sugar sensing pathways is complicated because photosynthesis and carbohydrate synthesis occur in both the mesophyll and bundle sheath cells instead of just the one cell type. Expression of genes encoding sugar sensor components could have evolved and been co-opted during C_4_ evolution to predominate in regulating sugars in the C_4_ source tissues. HXK is well known for its sugar feedback regulation of photosynthesis in the C_3_ dicot Arabidopsis, C_3_ monocot rice, and, to a lesser extent, in maize, a C_4_ monocot (Sheen, 1990; Moore *et al.*, 2003; Cho *et al.*, 2009). Electronic fluorescent pictographs (eFPs) depicting gene atlas expression from published RNA-seq data showed that the sensing HXK does not have preferential expression in source tissues over sink tissues of any of the three species depicted (Fig. 1). Preliminary analyses with available gene expression data and atlases across various tissues showed that there was no particular gene encoding sugar sensor components that was consistently expressed in the source tissues in either C_4_ or C_3_ species (Benning *et al.*, 2023). A gradient of sugar sensor gene expression was also not consistently observed for grasses as is observed for many sugar and starch metabolism genes during the transition from sink to source tissue along a developing leaf (Chen *et al.*, 2022). Protein sequence comparisons of C_4_ and C_3_ grasses across a phylogenetic spread showed that for many major sugar sensor components, the amino acid residue identity was often >90% (Table 1) (Benning *et al.*, 2023). This suggests that sugar sensors between C_4_ and C_3_ species are functionally very similar if not the same.

**Table 1.** Protein sequence identity comparison of some major sugar sensor components between the C_4_ grasses *Panicum antidotale*, *Sorghum bicolor*, *Setaria viridis*, and *Zea mays* and the C_3_ grasses *Cyrtococcum patens*, *Hymenachne amplexicaulis*, *Oryza sativa*, *Panicum bisulcatum*, and *Steinchisma laxum*

	PaHXK5	SbHXK5	SvHXK5	ZmHXK5	CpHXK5	HaHXK5	OsHXK5	PbHXK5	SlHXK5
PaHXK5		94.3	97.5	93.4	94.7	95	88.1	96.8	95.9
SbHXK5	94.3		96.1	96.6	95.4	95.9	88.3	96.6	96.8
SvHXK5	97.5	96.1		95.2	96.3	97	89.4	98.4	97.9
ZmHXK5	93.4	96.6	95.2		93.6	95.9	87.7	95.5	96.4
CpHXK5	94.7	95.4	96.3	93.6		95.6	89.4	96.3	96.6
HaHXK5	95	95.9	97	95.9	95.6		89.2	97	99.1
OsHXK5	88.1	88.3	89.4	87.7	89.4	89.2		89.4	89.4
PbHXK5	96.8	96.6	98.4	95.5	96.3	97	89.4		97.9
SlHXK5	95.9	96.8	97.9	96.4	96.6	99.1	89.4	97.9	
	PaHXK6	SbHXK6	SvHXK6	ZmHXK6	CpHXK6	HaHXK6	OsHXK6	PbHXK6	SlHXK6
PaHXK6		96.1	98.8	96.1	95.4	96.4	92.5	97.1	95.7
SbHXK6	96.1		96.4	97.8	94.2	95.4	91.6	94.7	95.2
SvHXK6	98.8	96.4		96.1	96.1	97.1	93	97.3	96.9
ZmHXK6	96.1	97.8	96.1		94.5	95.2	91.1	94.5	95.4
CpHXK6	95.4	94.2	96.1	94.5		94.7	91.8	94.5	94.2
HaHXK6	96.4	95.4	97.1	95.2	94.7		91.3	94.7	97.3
OsHXK6	92.5	91.6	93	91.1	91.8	91.3		92.1	91.8
PbHXK6	97.1	94.7	97.3	94.5	94.5	94.7	92.1		94.5
SlHXK6	95.7	95.2	96.9	95.4	94.2	97.3	91.8	94.5	
	PaSnRK1α1	SbSnRK1α1	SvSnRK1α1	ZmSnRK1α1	CpSnRK1α1	HaSnRK1α1	OsSnRK1α1	PbSnRK1α1	SlSnRK1α1
PaSnRK1α1		96.6	98	94.8	96.6	95.6	84.4	97.2	96.2
SbSnRK1α1	96.6		97.2	96.2	97.8	97	85.3	98.4	97.6
SvSnRK1α1	98	97.2		95.6	97	96	85	97.6	96.6
ZmSnRK1α1	94.8	96.2	95.6		96.4	96.6	85	97	97.4
CpSnRK1α1	96.6	97.8	97	96.4		97.4	84.8	98.4	97.8
HaSnRK1α1	95.6	97	96	96.6	97.4		84.8	97.6	98.6
OsSnRK1α1	84.4	85.3	85	85	84.8	84.8		85.5	85.1
PbSnRK1α1	97.2	98.4	97.6	97	98.4	97.6	85.5		98.4
SlSnRK1α1	96.2	97.6	96.6	97.4	97.8	98.6	85.1	98.4	
	PaTPS1	SbTPS1	SvTPS1	ZmTPS1	CpTPS1	HaTPS1	OsTPS1	PbTPS1	SlTPS1
PaTPS1		96.1	98	91.8	96.6	95.9	94	96.9	96
SbTPS1	96.1		96.9	94.7	96.2	96.7	93.6	96.8	96.4
SvTPS1	98	96.9		92.6	96.8	96.8	94.2	97.6	96.6
ZmTPS1	91.8	94.7	92.6		92	92.1	89.4	92.6	92.4
CpTPS1	96.6	96.2	96.8	92		96.2	93.6	96.8	96
HaTPS1	95.9	96.7	96.8	92.1	96.2		93.6	96.5	98.6
OsTPS1	94	93.6	94.2	89.4	93.6	93.6		93.5	93.3
PbTPS1	96.9	96.8	97.6	92.6	96.8	96.5	93.5		96.4
SlTPS1	96	96.4	96.6	92.4	96	98.6	93.3	96.4	

Note that for those species where there was no publicly available genome, a *de novo* assembly was performed which did not map small regions at the N- and C-terminus of the protein. Numbers represent percentage similarity.

Details on the mechanisms underpinning sugar sensing in C_4_ species remain elusive and at times conflicting. For example, in Setaria, a down-regulation of photosynthesis under high light is observed even when exposed for only 4 h at 900 μmol m^−2^ s^−1^ (Anderson *et al.*, 2021), but an up-regulation of photosynthesis under high light was observed when exposed for 4 d at 1000 μmol m^−2^ s^−1^ (Henry *et al.*, 2020). This highlights the importance of the difference between adaptation to treatments and an immediate response. Early work on maize indicated that this species was very tolerant to high foliar carbohydrate levels even when grown under continuous illumination, precluding diurnal starch breakdown (John *et al.*, 1973). Much of the evidence presented in this review suggests that sugar accumulation in C_4_ species can still cause a down-regulation in photosynthesis under certain conditions. Given the lack of cell-specific measurements of sugar levels in C_4_ leaves, it is difficult to ascertain whether treatments such as phloem loading perturbation and exogenous sugar feeding reflect physiologically relevant cellular sugar levels. In the case of sugarcane, the process of ‘ripening’, where sucrose accumulates in the stem following a reduction in growth (usually due to lower temperatures but also by plant growth regulators) but with continual provision of photosynthate from leaves, relies on photosynthesis proceeding even though sink strength has been reduced (Glasziou *et al.*, 1965). This would suggest a lack of sensitivity to sink feedback via sugar signalling in this C_4_ grass.

One of the major underlying questions is whether the accumulation and feedback regulation threshold is much higher in C_4_ leaves than in their C_3_ counterparts, whether dicot or monocot. Although difficult to achieve, examining the amount of sugars specifically in the mesophyll and bundle sheath cells of C_4_ species would also provide clarity on sugar sensing mechanisms. In the C_3_ grass barley, it has been estimated that the cytosol of the mesophyll cells have ~232 mM sucrose, while the parenchymatous bundle sheath can reach up to 100 mM under cold stress; however, this may in fact be much higher in C_4_ species and can change drastically under different conditions (Winter *et al.*, 1992; Koroleva *et al.*, 1998).

**Fig. 2.**
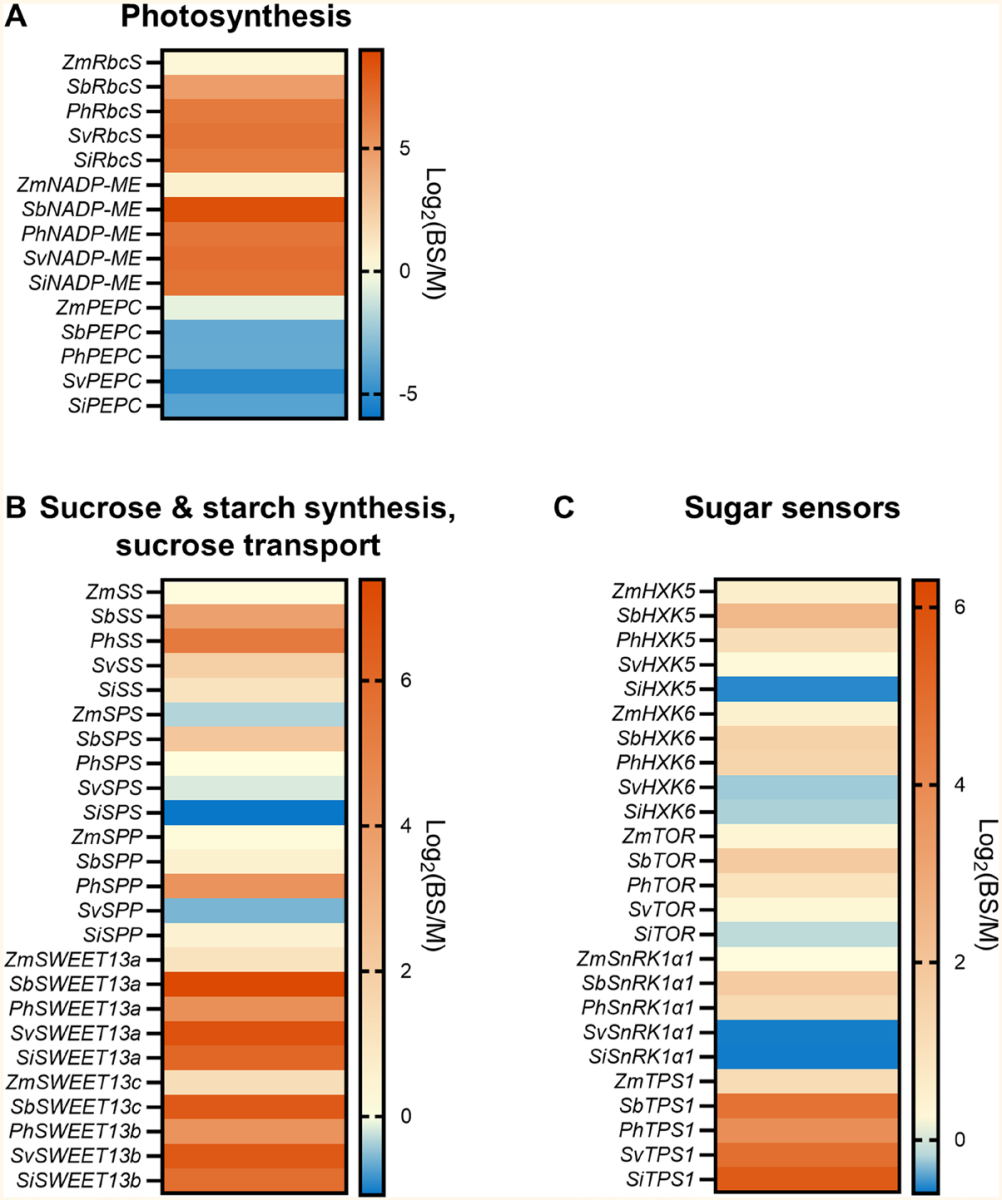
Heatmap expression of major genes encoding photosynthesis, sugar metabolism, and sensors in the bundle sheath and mesophyll cells. Gene expression from *Zea mays* (Zm) (Denton *et al.*, 2017), *Sorghum bicolor* (Sb) (Döring *et al.*, 2016), *Panicum hallii* (Ph) (Washburn *et al.*, 2021), *Setaria viridis* (Sv) (John *et al.*, 2014), and *Setaria italica* (Si) (Washburn *et al.*, 2021) bundle sheath and mesophyll cells. Expression of genes encoding Rubsico small subunit (RbcS), NADP-malic enzyme (NADP-ME), and phospho*enol*pyruvate carboxylase (PEPC) from the C_4_ photosynthetic pathway (A). Expression of genes encoding starch synthase (SS), sucrose phosphate synthase (SPS), sucrose phosphate phosphatase (SPP), and Sugars Will Eventually be Exported Transporters (SWEET) in the sucrose/starch synthesis and sucrose transport pathways (B). Expression of genes encoding components of sugar sensors hexokinase (HXK), Target of Rapamycin (TOR), Snf1-related kinase 1 (SnRK1) α1 subunit, and trehalose phosphate synthase 1 (TPS1) (C). Where there is more than one isoform present, the most abundantly expressed gene is depicted. The scale bar to the right of each heatmap depicts the log_2_ bundle sheath/mesophyll (BS/M) ratio where values >1 denote higher expression in bundle sheath, values <1 denote higher expression in mesophyll, and 0 denotes equal abundance in both photosynthetic cells.

Currently the specific roles that HXK, TORC1, SnRK1, and Tre6P play in regulating C_4_ photosynthesis remain unclear. Mechanical separation of mesophyll and bundle sheath cells is often used to determine their individual transcriptomes. Analyses of transcript abundance, protein abundance, and activity for photosynthetic and sugar/starch metabolism have been established between the bundle sheath and mesophyll cells (Lunn and Furbank, 1997, 1999;  Lunn, 2007; Furbank and Kelly, 2021). In the C_4_ grasses *Z. mays*, *Sorghum bicolor*, *Panicum hallii*, *Setaria viridis*, and *Setaria italica*, the genes encoding the Rubisco small subunit (RbcS) and NADP-ME and their expression predominates in the bundle sheath whereas *PEPC* is found in the mesophyll (see Box 1 for pathway) (Fig. 2A). This highlights the known compartmentalization of photosynthesis in C_4_ plants. While it was mentioned that sucrose and starch synthesis is often compartmentalized in C_4_ grasses, it can still vary from species to species (Lunn and Furbank, 1999). Starch synthesis has been more consistently found to occur in the bundle sheath cells, evidenced by transcriptomic, proteomic, and enzyme activity information (Furbank and Kelly, 2021). It is shown in Fig. 2B that the key starch synthesis gene, starch synthase, is more abundant in the bundle sheath for the each of the five C_4_ grasses. Genes encoding sucrose phosphate synthase (SPS) and sucrose phosphate phosphatase (SPP) within the sucrose biosynthesis pathway were more inconsistent in their expression between cells. Many *SPS* and *SPP* genes were equally abundant in both cell types except for *ZmSPS*, *SiSPS*, and *SvSPP* which seemed to be markedly more abundant in the mesophyll and *SbSPS* and *PhSPP* which were more abundant in the bundle sheath.  All C_4_ grasses showed a strong preferential expression of *SWEET13* genes in the bundle sheath cells. SWEET13 has been implicated to be an important export step for phloem loading in C_4_ grasses in the apoplastic pathway (Box 1) (Bezrutczyk *et al.*, 2018; Chen *et al.*, 2022; Hua *et al.*, 2022).

Analysis of transcript abundances of genes encoding sugar sensor components shows that there is some preferential expression of certain genes, but results are inconsistent between species (Benning *et al.*, 2023). Notably, *TPS1* was strongly expressed in the bundle sheath compared with mesophyll cells for all C_4_ grasses examined (Fig. 2C). TPS1 is part of the Tre6P signalling pathway (Box 1) where this metabolite is important for controlling diurnal starch degradation by signalling sucrose availability (Lunn *et al.*, 2006; dos Anjos *et al.*, 2018; Ishihara *et al.*, 2022). Therefore, in C_4_ species, it could be more functionally relevant if TPS was predominantly located in the bundle sheath to modulate Tre6P levels since this is the primary site of starch synthesis for many C_4_ grasses. When Tre6P levels were manipulated in maize, it was shown that this metabolite was important for regulation of photosynthesis, probably due to an increase in sink strength since *SWEET* expression also increased in reproductive organs (Oszvald *et al.*, 2018). While there has been evidence for Tre6P modulation of *SWEET* expression, the specific mechanisms behind this are yet to be resolved. Tre6P signalling-controlled expression of *SWEET13* in bundle sheath cells of C_4_ species could provide a regulatory response to sugar status at the first step of phloem loading.

Unlike the sensors such as kinases, which are limited in action to the cells where they are expressed, the metabolite Tre6P can potentially pass through the abundant plasmodesmatal connections between bundle sheath and mesophyll cells of C_4_ leaves, signalling photoassimlate abundance (Danila *et al.*, 2016). Tre6P could be important for sensing and controlling sucrose abundance in the bundle sheath, perhaps coordinating the flux of sucrose from the mesophyll, its synthesis after starch degradation from the chloroplast, and its export to the phloem for loading. Under high light in Setaria, as sucrose increases, levels of Tre6P also increase while total SnRK1 activity does not change when compared with low or medium light over 4 d (Henry *et al.*, 2020). *In vitro* Tre6P inhibition assays on SnRK1 activity show that activity was reduced by half, suggesting that this type of regulation via Tre6P and SnRK1 also occurs in C_4_ species. A comprehensive review of the role of Tre6P in crops by Paul *et al*. (2018) noted that Tre6P levels can be vastly different between Arabidopsis and crops such as wheat and maize. For example, Tre6P can reach up to 10 nmol g^–1^ FW in seedlings (Nunes *et al.*, 2013) and is typically lower in rosettes (Lunn *et al.*, 2006), whereas up to 119 nmol g^–1^ and 50 nmol g^–1^ FW of Tre6P has been detected in the wheat grain endosperm and maize kernel, respectively (Martínez-Barajas *et al.*, 2011; Nuccio *et al.*, 2015; Bledsoe *et al.*, 2017). Furthermore, the Tre6P:sucrose nexus appears to be weaker in these crops, suggesting that Tre6P may be different from the established mechanisms in Arabidopsis, possibly due to breeding for high yields (Martínez-Barajas *et al.*, 2011; Bledsoe *et al.*, 2017; Paul *et al.*, 2018). The Tre6P levels in the bundle sheath and mesophyll cells of C_4_ source leaves is unknown as is whether Tre6P is important for signalling related specifically to C_4_ photosynthesis.

*SnRK1α1* and *TOR* subunits show varied expression between the bundle sheath and mesophyll and between C_4_ species (Fig. 2) (see Benning *et al.*, 2023 for more detail). These master regulators are likely to be necessary in both bundle sheath and mesophyll cells of C_4_ species, possibly modulating photosynthetic activity in both cells, photoassimilate metabolism, and its subsequent transport. *HXK* sensor transcripts, however, were more abundantly expressed in the bundle sheath, except for *SiHXK5*, *SvHXK6*, and *SiHXK6*. The transcript abundance in the bundle sheath was more preferential for *TPS1* than for *HXK*, suggesting that in C_4_ grasses HXK is often present at similar levels in both the mesophyll and bundle sheath cells. This is possibly not surprising given that the known photosynthesis targets of HXK are housed in both the bundle sheath and mesophyll cells (Henry *et al.*, 2020).

Plant species also differ in their carbohydrate storage molecules, where some species use starch predominantly or sucrose in the vacuole which can be trafficked by SUTs, tonoplast-localized transporters (TSTs), or SWEETs depending on the gradient between the vacuole and cytosol. Setaria has been shown to contain some levels of fructans within their leaves (Chen *et al.*, 2022) which is more common in C_3_ temperate grasses such as barley and wheat (Pollock, 1986; Pollock and Cairns, 1991). Depending on the primary storage strategy of each plant, this could also result in differing sugar sensing pathways. Activity of sugar sensors and their signalling has also not been determined thus far for each photosynthetic cell type of C_4_ species. Although it must be noted that many of these sensors have more than one function or signalling role, isolating those functions related specifically to sugar sensing can be difficult in a single photosynthetic cell system of a C_3_ leaf, and even more complicated in a two-cell system of a C_4_ leaf.

AtHXK1 in Arabidopsis was one of the first sugar sensors whose sugar sensing function was separated from its catalytic function using *glucose insensitive* (*gin*) mutants where *HXK1* was knocked out and, as a result, could overcome high exogenous glucose application (Moore *et al.*, 2003). This seminal study established a direct link between photosynthesis regulation and a sugar sensor within a plant by using genetic manipulation of the gene encoding the protein of interest. This experiment has not been replicated in C_4_ plants to determine if similar phenotypes are observed.

The sensor and signal that coordinates sugar synthesis and export is still unknown in C_3_ and C_4_ plants. Earlier work on enzymes of sugar synthesis noted that they are relatively insensitive to product inhibition and so it is unlikely sucrose itself acts as a feedback signal (Koch *et al.*, 1996; Koch, 2004). As discussed, there is some level of feedback regulation on photosynthesis during sugar accumulation in C_4_ plants, but the mechanisms are also unknown. Given that C_4_ plants have substantially higher photosynthetic rates than most C_3_ plants (Byrt *et al.*, 2011), one would predict that rates of phloem export would need to be proportionally enhanced to fuel plant growth. Many C_4_ grasses follow the apoplastic pathway of phloem loading where there is active transport of sugar into the SE–CC complex via SUTs and passive export via SWEETs from phloem parenchyma and photosynthetic cells (Robinson-Beers *et al.*, 1990; Lohaus *et al.*, 1995; Slewinski *et al.*, 2010; Chen *et al.*, 2022). The efficiencies of this pathway compared with symplastic loading may prevent the build-up of photosassimilate at the point of export from the leaf, or C_4_ plants may be more tolerant to sugar accumulation. Currently, it is unclear which of these possibilities is correct. ^14^CO_2_-labelled gas exchange showed that while C_4_ plants showed a higher rate of photoassimilate export, the concurrent rate of export as a percentage of photosynthetic rate did not differ from C_3_ species, although this measurement does not accommodate remobilization of starch to sucrose and export at night (Grodzinski *et al.*, 1998). Therefore, it is possible that sugar transporters and diurnal export strategies have adapted to export needs of the efficient photosynthetic cells in a C_4_ system (Emms *et al.*, 2016).

## Conclusions

Sugar sensing and signalling in relation to the regulation of photosynthetic flux in the source leaf is not well known, especially in C_4_ plants, many of which are major cereal crops. Since improving photosynthetic performance has become a major frontier for increasing crop yields, it is pivotal to better understand the coordination of source and sink so that gains in photosynthetic capacity can be realized in increased yield (Reynolds *et al.*, 2012; Lawlor and Paul, 2014; Paul *et al.*, 2020). Current knowledge on sugar feedback regulation of photosynthesis has been established mainly in the C_3_ dicot Arabidopsis, but work in this model species should not be presumed to be directly translatable to a C_4_ monocot crop. The biochemical and anatomical specialization in C_4_ leaves required to support the two-cell photosynthetic mechanism has major ramifications for the regulation of carbon partitioning and sugar signalling, but almost nothing is known about these regulatory mechanisms in this important group of plants. Conflicting results of sugar feedback regulation that occurs in C_4_ plants means that the sugar sensing and signalling mechanisms remain ambiguous in these species. Sugar feedback probably does occur, but perhaps the mechanisms regulating it are different from those in C_3_ species given the anatomical complexities of C_4_ grasses and/or that the threshold which sugars levels must reach to elicit a response is higher.
